# Supplementary material for: Protective Effect of Colla corii asini against Lung Injuries Induced by Intratracheal Instillation of Artificial Fine Particles in Rats
Source: Int J Mol Sci. 2018 Dec 23;20(1):55. doi: 10.3390/ijms20010055 (PMC6337124; doi:10.3390/ijms20010055)
Supplement: Supplementary file 1 [file ijms-20-00055-s001.pdf]

International Journal of
Molecular Sciences

Supplementary File:

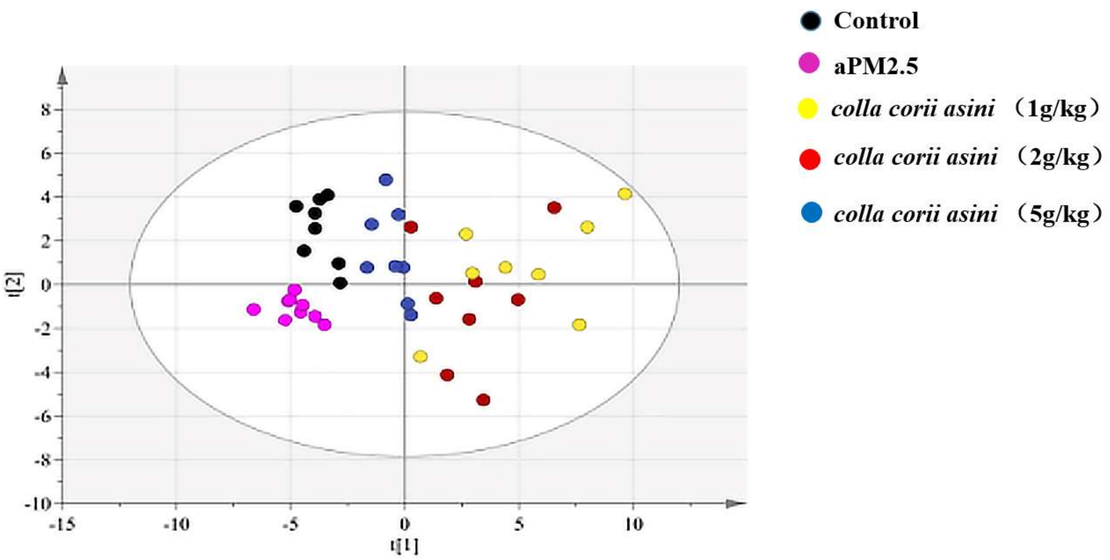

Figure S1. The PCA score plots of lung tissue from control, $aPM_{2.5}$, and *Colla corii asini* groups by LC-MS/MS analysis (n=8).

Table S1. The discriminating metabolites obtained from lung tissue samples of control, aPM2.5, and *Colla corii asini* groups by LC-MS/MS analysis. (n=8 per group).

Compound Name	Control	aPM2.5	*Colla corii asini*(1g/kg)	*Colla corii asini*(2g/kg)	*Colla corii asini*(5g/kg)	P value[a]	P value[b]	P value[c]	P value[d]
	Mean±SD	Mean±SD	Mean±SD	Mean±SD	Mean±SD				
O-Phospho-L-threonine	4.46E-03±1.44E-03	6.04E-03±8.01E-04	5.55E-03±1.44E-03	4.35E-03±1.98E-03	3.78E-03±2.69E-03	0.017	0.041	0.016	0.034
H-Homoarginine	4.62E-03±1.06E-03	7.25E-03±2.63E-03	6.44E-03±3.13E-03	7.27E-03±3.25E-03	7.52E-03±2.47E-03	0.027	0.593	0.888	0.852
L-O-Phosphoserine	3.11E-03±0.08E-04	3.70E-03±6.94E-04	4.07E-03±1.08E-04	5.12E-03±1.07E-04	5.40E-03±1.16E-04	0.057	0.443	0.007	0.007
L-Asparagine	0.951±0.24	1.108±0.25	0.936±0.29	0.865±0.17	0.883±0.30	0.229	0.242	0.044	0.161
L-Alanine	17.10±3.46	17.81±2.26	15.42±2.20	17.75±2.24	16.65±3.63	0.632	0.039	0.954	0.472
Hypotaurine	7.98E-02±2.83E-02	9.87E-02±2.66E-02	8.45E-02±2.17E-02	9.82E-02±3.04E-02	6.82E-02±2.35E-02	0.027	0.795	0.053	0.014
L-Aspartic acid	2.42±0.52	2.72±0.34	2.26±0.50	1.92±0.26	1.99±0.37	0.197	0.048	<0.001	0.003
L-cysteinesulfinic-acid monohydrate	2.16E-03±9.21E-04	1.92E-03±3.91E-04	2.47E-03±5.66E-04	2.64E-03±4.48E-04	2.64E-03±6.74E-04	0.522	0.041	0.027	0.051
L-Cysteine	0.14±0.029	0.18±0.059	0.25±0.050	0.25±0.049	0.16±0.077	0.054	0.025	0.029	0.486
L-Arginine	0.52±0.11	0.18±0.16	0.44±0.15	0.37±0.17	0.48±0.13	<0.001	<0.001	<0.001	<0.001
L-Histidine	0.152±0.049	0.160±0.030	0.164±0.078	0.321±0.059	0.329±0.108	0.811	0.802	<0.001	<0.001
4-Hydroxy-pyrrolidine-2-carboxylic	3.13E-02±6.04E-03	2.32E-02±4.16E-03	2.62E-02±2.15E-03	2.74E-02±3.13E-03	2.82E-02±2.95E-03	0.045	0.043	0.042	0.047
L-Glutamic acid	5.91±1.39	6.15±0.91	5.70±0.92	4.94±0.74	5.03±1.03	0.682	0.333	0.011	0.050
Pipecolic acid	3.26E-02±4.24E-03	5.95E-02±3.98E-03	4.68E-02±7.25E-03	4.11E-02±5.53E-03	4.55E-02±2.25E-03	0.016	0.074	0.065	0.070
Saccharopine	2.31E-03±3.03E-04	2.41E-03±2.43E-04	2.49E-03±6.00E-04	3.14E-03±6.32E-04	4.49E-03±4.72E-04	0.507	0.706	0.009	0.070
DL-Homocysteine	9.77E-03±2.71E-03	9.82E-03±1.83E-03	1.25E-02±3.29E-04	1.34E-02±2.71E-04	1.27E-02±2.33E-04	0.966	0.225	0.018	0.022
γ-AminobutyrIec Acid	5.00E-02±1.40E-02	6.34E-02±1.82E-02	7.54E-02±0.49E-02	1.15E-01±1.88E-02	8.754E-02±1.81E-02	0.122	0.302	<0.001	0.030

L-Isoleucine	16.59±3.69	19.51±4.32	22.27±4.82	19.39±3.56	20.09±4.46	0.167	0.043	0.950	0.815
L-Lysine	6.21.10±2.19	7.27±1.64	5.32±1.67	7.59±1.76	7.87±0.89	0.165	0.026	0.714	0.446
L-Leucine	30.25±6.30	35.68±6.52	33.02±3.20	38.87±6.62	42.82±2.56	0.113	0.452	0.348	0.039
L-Homoserine	2.56E-02±1.46E-02	2.87E-02±2.54E-02	3.47E-02±2.79E-02	6.84E-02±3.94E-02	9.19E-02±8.61E-02	0.759	0.674	0.031	0.070
L-Methionine	7.24±1.85	7.86±3.05	7.84±2.02	9.46±1.73	10.64±2.23	0.539	0.986	0.112	0.045
L-Citrulline	4.27E-02±9.86E-03	3.79E-02±0..81E-03	3.917E-02±9.90E-03	3.79E-02±1.57E-03	4.95E-02±9.40E-03	0.303	0.780	0.984	0.027*

Note: To calculate the statistical significance *P* value[a] (control group versus $aPM_{2.5}$ group), *P* value[b] ($aPM_{2.5}$ group versus *Colla corii asini* (1g/kg) group) *P* value[c] ($aPM_{2.5}$ group versus *Colla corii asini* (2g/kg) group), *P* value[d] ($aPM_{2.5}$ group versus *Colla corii asini* (5g/kg) group), Shapiro-Wilk test was first used to verify the normality of data. When the data were normally distributed, statistical significance was determined using two-side t-test; otherwise, significance was determined by Nonparametric tests (*). Data were calculated from the ratio of the mean peak areas in the control, $aPM_{2.5}$, and *Colla corii asini* groups and normalized to L-norvaline (internal standard).
